# Skel-Net: automatic prediction of skeletal pattern on scanned lateral cephalograms using anatomical prior-guided deep learning network

**DOI:** 10.1186/s12903-025-06771-z

**Published:** 2025-10-31

**Authors:** Eun Sun Song, Su Yang, Won-Jin Yi, Seung-Pyo Lee

**Affiliations:** 1 Department of Oral Anatomy, School of Dentistry, Seoul National University, Seoul, Republic of Korea; 2 Department of Applied Bioengineering, Graduate School of Convergence Science and Technology, Seoul National University, Seoul, Republic of Korea; 3 Department of Oral and Maxillofacial Radiology, School of Dentistry, Seoul National University, Seoul, Republic of Korea

**Keywords:** Skeletal malocclusion, Cephalometric analysis, Deep learning

## Abstract

**Background:**

Estimating craniofacial patterns is essential for successful orthodontic treatment. However, conventional static measurements are inadequate for capturing dynamic changes, and manual cephalometric analysis is labor-intensive and requires specialized expertise. In this study, we propose Skel-Net, a novel anatomic prior-guided deep learning network designed to estimate ANB angle changes over five years in children and adolescents aged 8–16.

**Methods:**

In a two-stage approach, Skel-Net combines cephalometric landmark detection via Ceph-Net and multichannel inputs, including two-dimensional heatmaps and ANB priors, to enhance prediction accuracy. A dataset of 612 lateral cephalograms from 245 patients was used to train and validate the model, and its performance was compared against DenseNet121, MobileNetV2, ResNet101, and VGG16.

**Results:**

Skel-Net outperformed the other models with the lowest prediction errors (mean absolute error: 1.021 degrees; root mean squared error: 1.338 degrees) and the highest *R*^2^ value (0.517), demonstrating robust predictive capabilities.

**Conclusions:**

By leveraging anatomic priors and longitudinal data, Skel-Net enables dynamic and personalized predictions of craniofacial growth. This framework will facilitate early and precise orthodontic interventions, enhancing treatment efficiency, stability, and overall patient outcomes.

## Background

Skeletal malocclusion, the abnormal alignment of the dental arch, is usually the result of an anterior-posterior jaw imbalance [1]. This condition affects critical oral functions, including chewing and speech. It also influences aesthetic, social, and psychological well-being, particularly vital for children and adolescents, whose self-image and social development can be detrimentally affected, leading to challenges in interpersonal relationships and social interactions. Skeletal malocclusion, once established in early childhood, rarely self-corrects during development [2, 3]. Even in class III cases, the condition worsens over time [4, 5]. Therefore, predicting craniofacial growth patterns and initiating timely and effective treatment is crucial to enhancing orthodontic outcomes. However, making those predictions is inherently complex because of genetic and environmental influences and individual growth variations [6, 7]. To address this challenge, we propose Skel-Net, a deep learning-based framework for predicting changes in the ANB angle five years into the future. This novel approach could inform clinical decision-making and improve treatment stability.

Longitudinal data are indispensable for understanding the natural progression of malocclusions and the effectiveness of various treatment strategies [8]. Capturing developmental changes in craniofacial structures throughout growth is fundamental to optimizing orthodontic treatment planning during the crucial growth phases of childhood and adolescence. Unlike cross-sectional studies, which capture a condition at a single point, longitudinal studies track the same individuals over extended periods [9]—an invaluable approach that offers insights into each individual’s growth process and patterns, significantly influencing treatment planning and outcomes. Kim et al. [9] proposed a study using longitudinal lateral cephalometric radiographs to evaluate various machine learning models for accurately predicting craniofacial growth in a Japanese cohort. Their results demonstrated that the least absolute shrinkage and selection operator (LASSO) model achieved the highest prediction accuracy for craniofacial growth, with accuracies of 97.87% for linear parameters and 94.45% for angular parameters. The study highlighted that LASSO effectively minimized prediction errors for skeletal landmarks linear and angular parameters. However, despite its accuracy, the study acknowledged limitations in handling complex growth patterns and emphasized the need for models that could further enhance prediction reliability. Taloumtzi et al. [10] focused on changes in the maxillomandibular relationships from childhood to adolescence in enrollees with class II malocclusion, revealing that significant profile straightening occurs during growth, particularly in individuals with an initially increased overjet. That study highlighted the natural progression of malocclusions and the potential for spontaneous improvements over time, processes that are critical for timing orthodontic interventions. Recent studies have shown increased interest in leveraging AI models for predicting craniofacial growth based on serial cephalometric data. Larkin et al. [11] applied convolutional neural networks to forecast landmark positions two years into the future in preadolescents, demonstrating moderate accuracy for most points, particularly in hard-tissue regions. Moreover, Larkin et al. emphasized that anatomically interpretable and clinically guided AI architectures are essential for meaningful integration into orthodontic practice. These recent findings highlight both the potential and the limitations of current AI-based growth prediction systems. In this context, the present study contributes to the field by proposing a two-stage deep learning network that incorporates anatomical priors to predict ANB angle changes over a five-year horizon. Despite the numerous studies using longitudinal data ranging from growth prediction to understanding the history of dental and skeletal anomalies, few have focused on predicting future skeletal patterns or validating such predictions with follow-up data from the same patients.

Skeletal malocclusion is classified using various angular and linear measurements from lateral cephalograms. The present study focused on the ANB angle as the primary indicator for assessing skeletal relationships. The ANB angle, which stands for the A, nasion (N), and B points, is measured on lateral cephalometric images to evaluate the anterior-posterior relationship between the maxilla and mandible [12, 13]. This measurement is essential for assessing the sagittal skeletal relationship and is closely related to the occlusal relationship and facial appearance. The ANB angle has long been recognized as a gold standard in cephalometric analysis for evaluating sagittal skeletal discrepancies due to its simplicity and clinical relevance. Multiple studies have confirmed its strong correlation with other sagittal indicators such as the Wits appraisal and Beta angle, validating its continued use in both clinical and AI-based diagnostic settings [14, 15].

Skeletal malocclusion classification has traditionally involved manual measurements, a time-consuming and labor-intensive process with limitations in tracking dynamic craniofacial growth changes. Advances in artificial intelligence and deep learning have significantly transformed various fields of medicine and dentistry, driving progress in diagnosis and treatment [16–18]. In particular, several studies evaluating the effectiveness of artificial intelligence for estimating craniofacial growth patterns have begun to address the time and labor challenges. Wood et al. [19] used machine learning techniques to predict mandibular length and Y-axis growth in male enrollees accurately. Their retrospective longitudinal study reported that accuracy ranged from 95.80 to 97.64% for mandibular length predictions and 96.60–98.34% for Y-axis growth predictions. While the models demonstrated promising predictive capabilities, incorporating anatomical and growth-related variables could enhance accuracy. The study also emphasized the importance of refining model input variables and utilizing longitudinal data for improved predictions. Zhang et al. [20] used a ResNet50-based deep learning model to predict mandibular growth trends in children with an anterior crossbite, achieving superior accuracy compared with predictions made by junior orthodontists. Furthermore, several studies have explored the use of deep learning models for automatic skeletal classification in lateral cephalograms. Yu et al. [21] proposed a multimodal convolutional neural network that integrates demographic data for skeletal classification, achieving 96.40% accuracy. Kim et al. [22] reported the effectiveness of a deep convolutional neural network–based artificial intelligence model for classifying sagittal skeletal relationships that outperformed automated tracing software in various performance metrics. However, that research has chiefly targeted adult populations and focused on identifying skeletal patterns without considering the longitudinal changes essential for a comprehensive understanding of craniofacial development. Therefore, methods to infer growth patterns during key growth stages in children and adolescents represent a significant need for successful orthodontic treatment outcomes.

To address these limitations, our study offers three key contributions. First, we utilize longitudinal cephalometric data from children and adolescents aged 8–16, enabling the model to learn from real-time skeletal growth trajectories over five years, a dataset and age group rarely used in previous work. Second, we design a two-stage deep learning framework, where heatmaps of anatomical landmarks and ANB priors are used as multichannel inputs to guide anatomically structured learning. Third, unlike prior classification-focused approaches, our model predicts continuous changes in the ANB angle five years into the future, allowing for dynamic and personalized skeletal growth prediction.

Recent advancements in deep learning have led to an increasing use of AI in craniofacial growth prediction. Several studies have utilized convolutional neural networks (CNNs) or hybrid models to predict skeletal class or growth trends from cephalometric images, demonstrating promising accuracy and clinical applicability [23].

In the present study, we propose an anatomic prior-guided deep learning network called Skel-Net that predicts skeletal malocclusion patterns in a two-stage process by estimating ANB angle changes five years into the future in children and adolescents aged 8–16. First, Ceph-Net automatically detects cephalometric landmarks and generates corresponding two-dimensional (2D) heatmaps. Second, Skel-Net uses those 2D heatmaps and ANB priors alongside digitized lateral cephalograms as multichannel inputs to predict future changes in the ANB angle. By leveraging the 2D heatmaps and ANB priors, Skel-Net effectively captures anatomic structures and cephalometric patterns to predict skeletal malocclusion patterns accurately.

## Methods

### Data acquisition and preparation

The dataset for this study consisted of 612 lateral cephalograms from 245 patients (mean age: 11.9 years; age range: 8–16 years; 117 girls and young women, 128 boys, and young men) who underwent oral examination at the Seoul National University School of Dentistry, Korea, from 1995 to 2003. We categorized the patients into two age groups for analysis: 8–11 years (G1) and 13–16 years (G2). The inclusion criteria were age between 8 and 16 years, availability of lateral cephalograms taken five years apart, and no orthopedic or orthodontic treatment history. The lateral cephalograms allocated to the training, validation, and test data subsets were 385, 129, and 98, respectively. A film scanner (Epson Perfection V850 Pro: Seiko Epson Corp., Tokyo, Japan) was used to digitize the lateral cephalograms at 300 dpi and export the resulting images in TIF format for analysis. The scanned images (2400 × 3000 px) were resized to 576 × 736 px based on the size used in a previous study. Ethical approval for this study (S-20210028) was obtained from the Seoul National University Graduate School of Dentistry’s Research Ethics Committee. The requirement for informed consent was waived, given the study’s retrospective nature involving anonymized data, and all procedures adhered to the relevant guidelines and regulations.

Following digitization, a clinically experienced dentist manually annotated cephalometric landmarks using the Labelbox software application (Labelbox Inc., San Francisco, CA, USA). These manually annotated landmarks served as the ground truth dataset for training the Ceph-Net model, which was later used for automatic landmark detection. The ANB angle between the A, nasion (N), and B points defines the sagittal relationship between the maxilla and mandible. In this study, the digitized cephalograms were allocated to class I, II, or III based on the value of the ANB angle (class I: 3.2–5.7 degrees; class II: >5.7 degrees; class III: < 3.2 degrees [24]).

### Patient demographics and the test set

The 98 digitized cephalograms allocated to the test set were categorized as follows: class I, 24 cephalograms (11 from male patients, 13 from female patients; mean age: 9.5 years; average ANB angle: 4.397 degrees); class II, 9 cephalograms (all from female patients; mean age: 9.44 years; average ANB angle: 7.011 degrees); class III, 65 cephalograms (27 from male patients, 38 from female patients; mean age: 9.58 years; average ANB angle: 1.703 degrees). A class-weighting approach was applied to the loss function to address class imbalance during training, assigning higher weights to underrepresented classes (Class I and II) to ensure balanced learning. Data augmentation techniques were applied uniformly across all classes to enhance model robustness and reduce overfitting. Those demographic distributions were used to analyze craniofacial growth trends and to assess the accuracy of the deep learning models in predicting skeletal malocclusions (Table 1).

**Table 1:** Summary of patient demographics and skeletal class distribution in the test dataset

Skeletal class	Patients (*n*)			Mean age (years)	Mean ANB angle (degrees)
	Overall	Male	Female		
I	24	11	13	9.50	4.397
II	9	0	9	9.44	7.011
III	65	27	38	9.58	1.703

**Fig. 1:**
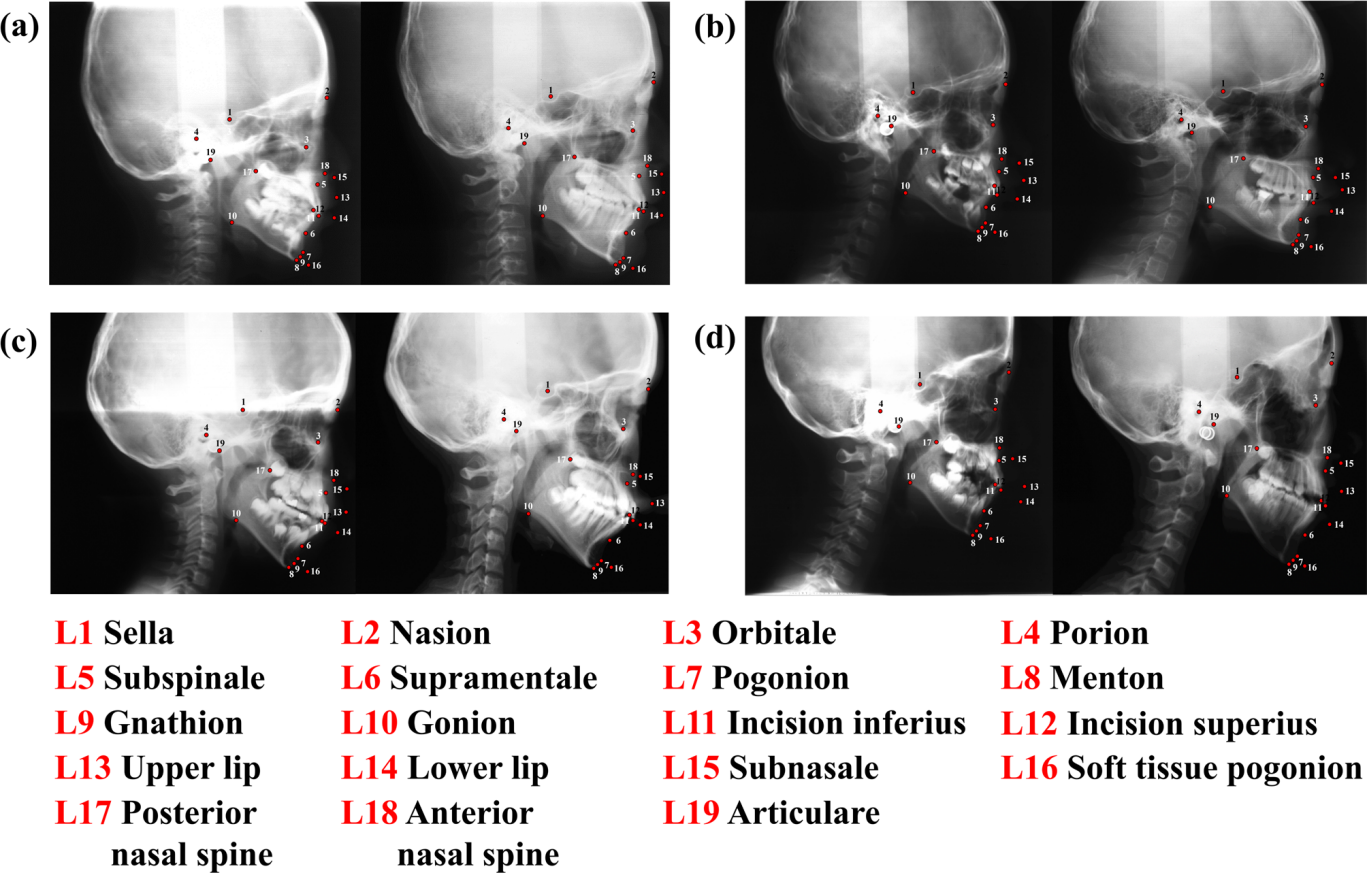
Lateral cephalograms were derived from longitudinal data obtained with the same individual at five-year intervals. **a** At age 8 and 13 years. **b** At age 9 and 14 years. **c** At age 10 and 15 years. **d** At age 11 and 16 years. L1, sella; L2, nasion; L3, orbitale; L4, porion; L5, subspinale; L6, supramentale; L7, pogonion; L8, menton; L9, gnathion; L10, gonion; L11, incision inferius; L12, incision superius; L13, upper lip; L14, lower lip; L15, subnasale; L16, soft tissue pogonion; L17, posterior nasal spine; L18, anterior nasal spine; L19, articulare

Figure 1 presents examples of lateral cephalograms that illustrate skeletal development over time. Those paired cephalograms, taken at ages 8–13, 9–14, 10–15, and 11–16, indicate how craniofacial structures change during key developmental phases in children and adolescents. Consistent five-year intervals provided longitudinal data for detailed analysis of craniofacial growth patterns, enabling practical training and evaluation of the proposed deep learning model.

### The network architecture of Skel-Net

**Fig. 2:**
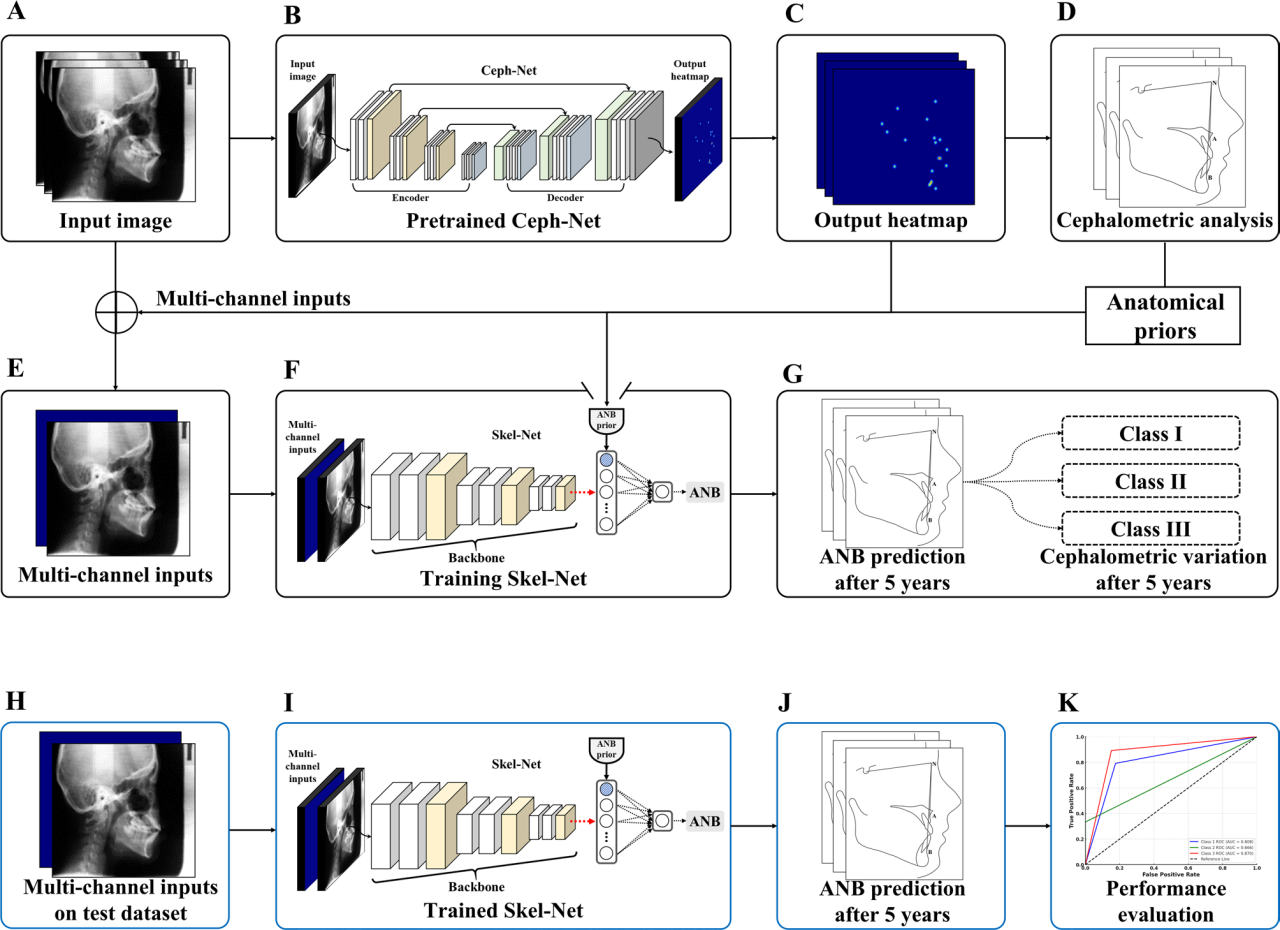
The proposed method. **a** Lateral cephalograms were obtained using the training set. **b** Apply pretrained Ceph-Net. **c** Two-dimensional output heatmaps of 19 cephalometric landmarks. **d** Cephalometric analysis based on the output heatmaps. **e** Multichannel inputs. **f** Training Skel-Net. **g** ANB angle prediction after five years (training set). **h** Multichannel inputs from the test set. **i** Apply trained Skel-Net. **j** ANB angle prediction after five years (test set). **k** Skel-Net performance evaluation on the test set

The proposed framework has two stages: Ceph-Net [25] for cephalometric landmark detection and Skel-Net for predicting skeletal malocclusion patterns (Fig. 2). In the first stage, the 19 cephalometric landmarks are automatically detected in the digitized cephalograms by Ceph-Net, a landmark detection model developed during our earlier work. Ceph-Net generates 2D heatmaps of the landmarks that are used as inputs, together with the digitized cephalograms, in the second stage (Skel-Net) for predicting skeletal malocclusion patterns. Skel-Net then predicts the ANB angle five years into the future based on those multichannel inputs.

**Fig. 3:**
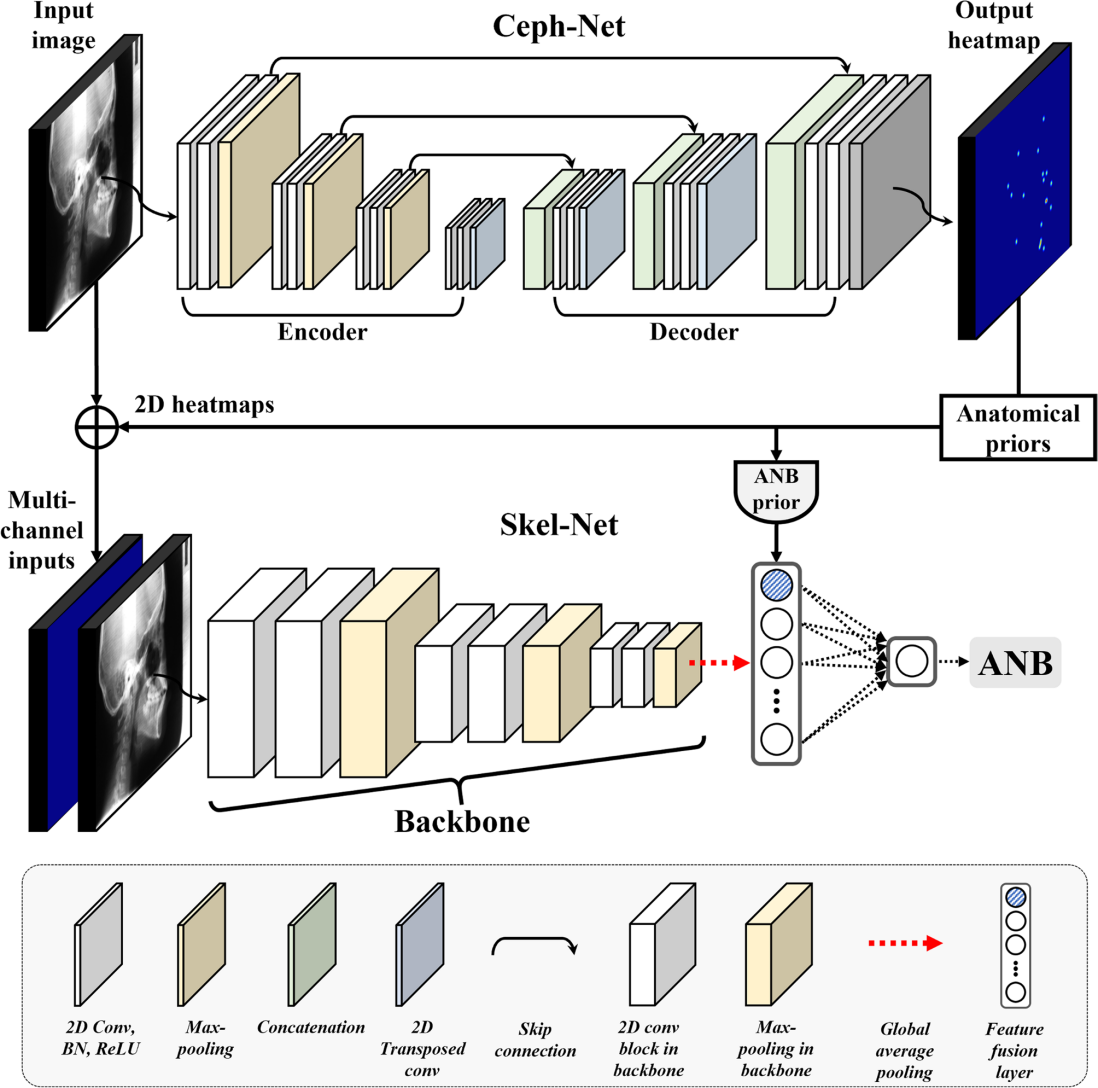
The proposed framework has two stages: Ceph-Net for cephalometric landmark detection and Skel-Net for predicting skeletal malocclusion patterns. 2D, two-dimensional

Skel-Net is an anatomic prior-guided deep learning network that consists of a 2D backbone, multichannel inputs, and ANB priors (Fig. 3). The multichannel inputs integrate the digitized cephalograms with the corresponding 2D heatmaps generated by Ceph-Net to provide anatomic priors, which are then used to simultaneously learn both the anatomic structure and the initial skeletal patterns. Convolutional neural network–based backbones (VGG16 [26], ResNet101 [27], DenseNet121 [28], MobileNetV2 [29], and EfficientNet-B4 [30]) were used to extract the anatomic features of the skeletal patterns at the encoder. To enhance the ability of Skel-Net to recognize skeletal patterns, the ANB priors computed by Ceph-Net were integrated with flattened feature maps obtained through global average pooling of anatomic features. The ANB prior is defined as the angle between the vectors formed by the anatomical landmarks of A point (A), Nasion (N), and B point (B) [25] detected from Ceph-Net. Specifically, it is calculated as the angle between vectors NA and NB, where A and B represent the maxillary and mandibular points, respectively, and N serves as the vertex. This angle reflects the geometric relationship between the maxilla (A point) and the mandible (B point) relative to the cranial base (Nasion). The ANB prior provides a clinically meaningful representation of the baseline skeletal relationship between the maxilla and mandible, serving as a critical temporal reference for predicting future skeletal changes. Finally, a linear activation function is used to predict the ANB angle five years into the future. We used the MSE to train Skel-Net. The MSE measures the average error of the squared differences between a prediction and the ground truth:

1 $$\:\text{M}\text{S}\text{E}\left(y,p\right)\:=-\frac{1}{N}\sum \limits _{i=1}^{N}{{(y}_{i}-{p}_{i})}^{2}$$

where *N*,* y*, and *p* are, respectively, the number of samples, the ground truth, and the prediction result.

The proposed Skel-Net model was trained for 200 epochs with a mini-batch size of four on a workstation with an Intel i9-7900X CPU operating at 3.3 GHz, 128 GB RAM, and two Nvidia GeForce GTX 1080 Ti graphics processing units. Data augmentation was performed with random rotation (− 10 to 10 degrees), width and height shift (− 5–5%) of the image size in the horizontal and vertical axes, zoom (− 5–5%), and Gaussian noise (*µ* = 0.0 and *σ*^2^ = [0,0.1]). The Adam optimizer was used with a learning rate of 10^−3^. The learning rate was gradually reduced by half up to 10^−6^ when validation loss saturated for 25 epochs. The proposed Skel-Net was implemented in Python 3 using the Keras framework with the TensorFlow backend.

### Evaluation metrics

The metrics used to evaluate and analyze model performance were the MAE, MSE, RMSE, and *R*².

The MAE calculates the average magnitude of the absolute difference between the predicted and actual ANB angles. This intuitive metric provides an understanding of how closely the predicted ANB angle aligns with the actual angle:

2 $$\:\text{M}\text{A}\text{E}=\:\frac{1}{n}\sum\limits_{i=1}^{n}|{P}_{i}-{R}_{i}|$$

where *P*_*i*_ is the predicted value for the *i-*th observation, and *R*_*i*_ is the corresponding actual value.

The MSE calculates the mean of the squared difference between the predicted and actual ANB angles. Squaring the residuals penalizes larger errors more heavily, making this metric highly sensitive to outliers:

3 $$\:\text{M}\text{S}\text{E}=\:\frac{1}{n}\sum\limits_{i=1}^{n}{({P}_{i}-{R}_{i})}^{2}$$

The RMSE is the square root of the MSE, providing an error measure in the same units as the ANB angle. The RMSE is thus easier to interpret and more relevant for assessing prediction accuracy:

4 $$\:\text{R}\text{M}\text{S}\text{E}=\sqrt{\frac{1}{n}\sum\limits_{i=1}^{n}{({P}_{i}-{R}_{i})}^{2}}$$

The *R*² metric quantifies the proportion of variance in the actual ANB angle that the model can explain. A value closer to 1 indicates a better fit and stronger predictive alignment:

5 $$\:{R}^{2}=1\:-\:\frac{{\sum\:}_{i=1}^{n}({{R}_{i}-{P}_{i})}^{2}}{{\sum\:}_{i=1}^{n}({{R}_{i}-\stackrel{-}{R})}^{2}}$$

where $\:\stackrel{-}{R}$ is the mean of the actual angle.

### Statistical analysis

To evaluate the significance of the differences in MAE between Skel-Net and each baseline model, we conducted pairwise statistical tests. Before hypothesis testing, the normality of the paired differences was assessed using the Shapiro–Wilk test (*p* > 0.05 indicates normality). When the assumption of normality was satisfied, paired t-tests were performed; otherwise, the Wilcoxon signed-rank test was applied. Based on this approach, the comparison between Skel-Net and VGG16 was analyzed using the Wilcoxon signed-rank test, whereas the other comparisons employed paired t-tests. Statistical significance was defined as *p* < 0.05. All statistical analyses were performed using IBM SPSS Statistics (version 29.0; IBM Corp., Armonk, NY, USA).

## Results

As shown in Table 2, Skel-Net achieved the lowest regression errors (MAE: 1.021°, RMSE: 1.338°) and the highest *R*² value (0.517), indicating solid performance in predicting ANB angle changes. These results align with the model’s classification performance—Skel-Net attained the highest accuracy (81.6%) and precision (83.8%) among all models. In particular, the confusion matrix (Fig. 6) shows strong discrimination in classifying skeletal patterns, especially for class III. This consistency across both regression and classification metrics highlights the model’s overall robustness and potential clinical utility. As shown in Table 3, the integration of ANB priors and heatmaps progressively improved the model’s performance across all metrics. These results demonstrate the complementary benefits of anatomical priors and 2D heatmaps in enhancing longitudinal ANB prediction. In Table 4, applying data augmentation to Skel-Net consistently improved performance across all evaluation metrics.

**Table 2:** Comparative performance of deep learning models for predicting ANB angle and skeletal class

Model	MAE (degrees)	MSE (degrees)	RMSE (degrees)	*R* ^2^	Accuracy (%)	Precision (%)	Recall (%)	F1 score (%)
Skel-Net	1.021	1.791	1.338	0.517	81.6	83.8	67.2	69.5
DenseNet121	1.075	1.930	1.390	0.491	79.6	75.9	67.7	69.9
MobileNetV2	1.048	1.913	1.383	0.491	77.6	80.0	59.9	64.3
ResNet101	1.090	1.993	1.412	0.474	77.6	80.4	59.4	61.4
VGG16	1.171	2.160	1.470	0.447	74.5	78.6	57.8	59.5

*MAE* mean absolute error, *MSE* mean squared error, *RMSE* root mean squared error

**Table 3:** Performance metrics of the models for each component. The baseline with ANB prior and heatmaps indicates our Skel-Net

Components	MAE (degrees)	MSE (degrees)	RMSE (degrees)	*R* ^2^	Accuracy (%)	Precision (%)	Recall (%)	F1 score (%)
Baseline	1.070	2.075	1.440	0.490	74.5	77.5	52.0	52.8
+ ANB prior	1.071	1.904	1.380	0.489	77.5	81.4	65.2	66.8
+ Heatmaps	1.073	1.895	1.377	0.503	76.5	81.3	68.8	69.9
+ ANB prior and Heatmaps	1.021	1.791	1.338	0.517	81.6	83.8	67.2	69.5

*MAE* mean absolute error, *MSE* mean squared error, *RMSE* root mean squared error

**Table 4:** Performance metrics of the models for data augmentation (DA)

Components	MAE (degrees)	MSE (degrees)	RMSE (degrees)	*R* ^2^	Accuracy (%)	Precision (%)	Recall (%)	F1 score (%)
Skel-Net w/o DA	1.055	1.942	1.393	0.476	75.5	79.1	61.5	64.4
Skel-Net w/DA	1.021	1.791	1.338	0.517	81.6	83.8	67.2	69.5

*MAE* mean absolute error, *MSE* mean squared error, *RMSE* root mean squared error

To further evaluate whether the observed differences in prediction error were statistically significant, we performed pairwise statistical tests between Skel-Net and each baseline model. As summarized in the revised Table 5, only the comparison between Skel-Net and ResNet101 yielded a statistically significant difference in MAE (*p* = 0.029), whereas the other comparisons were not statistically significant (all *p* > 0.05).

**Table 5:** Statistical comparison of prediction errors (MAE) between Skel-Net and baseline models

Baseline Model	Statistical Test	*p*-value
DenseNet121	Paired t-test	0.146
MobileNetV2	Paired t-test	0.412
ResNet101	Paired t-test	0.029*
VGG16	Wilcoxon signed-rank test	0.738

*p < 0.05, statistically significant difference between Skel-Net and ResNet101

**Fig. 4:**
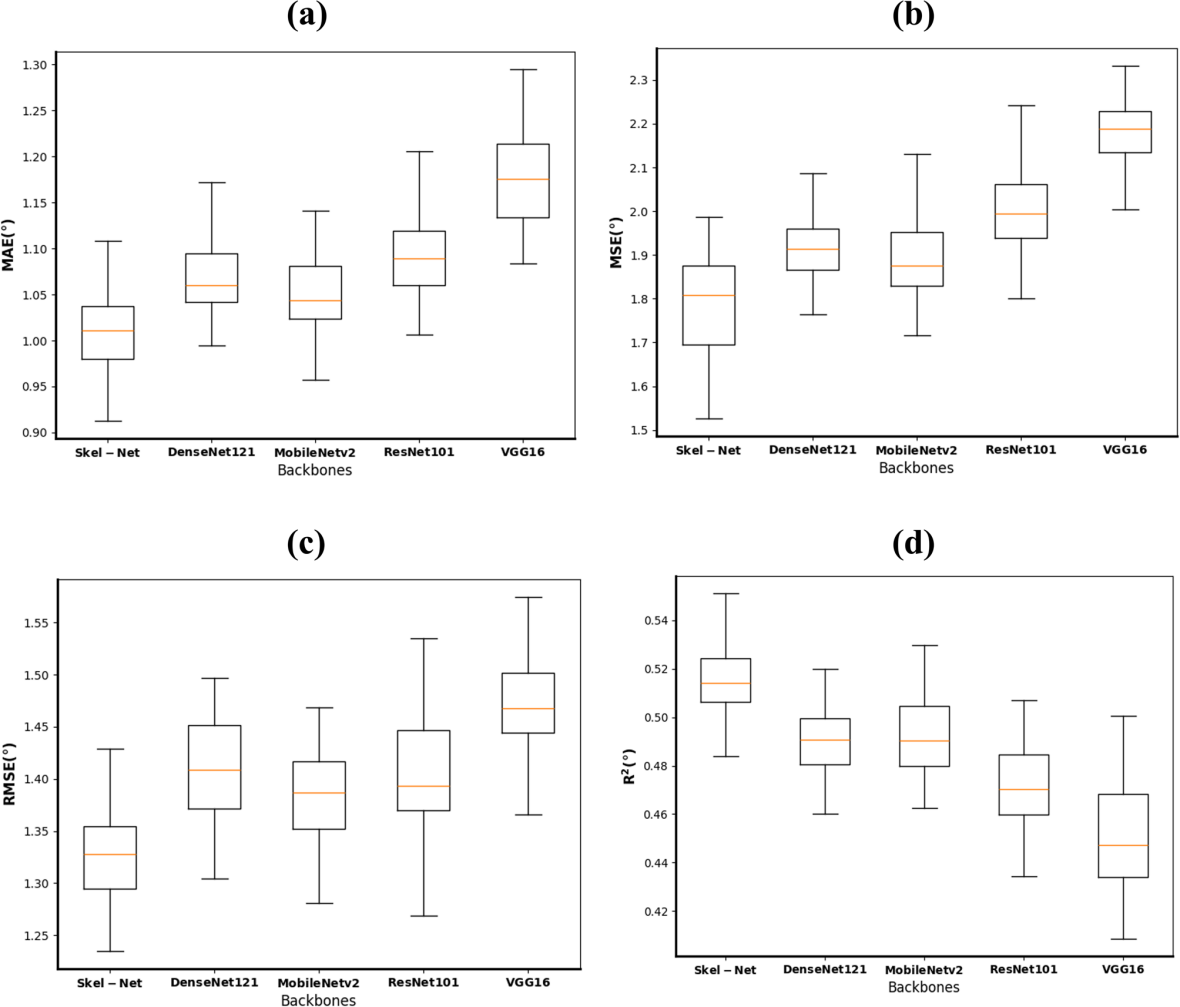
Box plots of performance metrics (MAE, MSE, RMSE, R²) for skeletal malocclusion prediction using different model backbones

Figure 4 uses box plots for each metric (MAE, MSE, RMSE, *R*^2^) to display the distribution of prediction errors for the various models. The plots reveal that Skel-Net has a more concentrated and lower range of error values across all metrics, reinforcing its performance advantage over the other models. DenseNet121 and MobileNetV2 had broader distributions and slightly higher error rates.

**Fig. 5:**
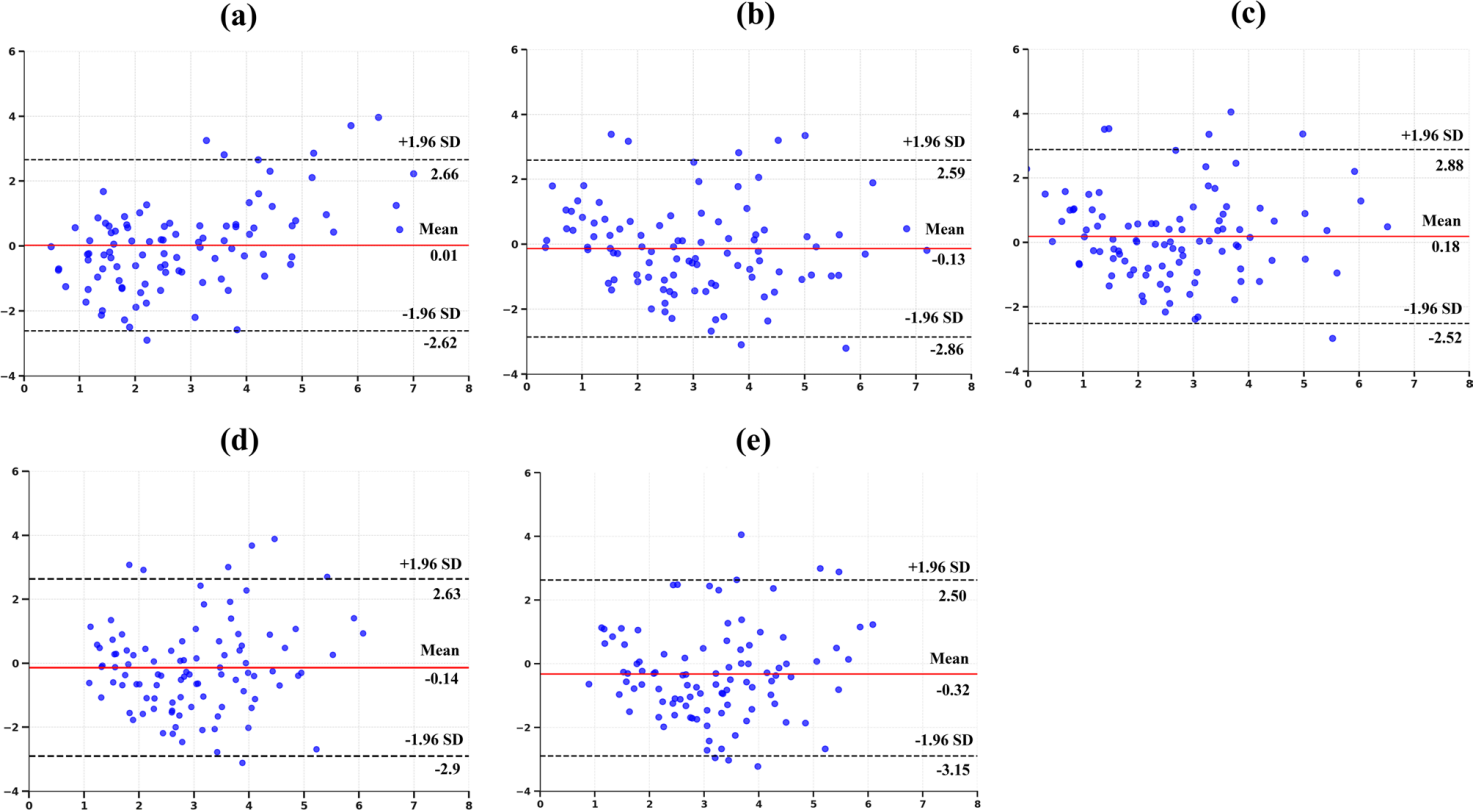
Bland–Altman plots comparing predicted and actual ANB angles across different models. **a** Skel-Net. **b** DenseNet121. **c** MobileNetV2. **d** ResNet101. **e** VGG16. SD, standard deviation

Figure 5 uses Bland–Altman plots to compare the agreement between predicted and actual ANB angles for all models. In those plots, Skel-Net demonstrated the narrowest limits of agreement of all the models, suggesting better predictive accuracy and lower bias. Specifically, the distribution of differences was more tightly clustered around the zero-difference line with Skel-Net, implying that its predictions were consistently closer to the actual values. Although a few data points exhibited some variability and outliers, the overall distribution was still more tightly clustered than other models. In contrast, models such as MobileNetV2 and ResNet101 had a wider spread of differences and more data points falling outside the limits of agreement, indicating less precise predictions with increased variability.

To further evaluate the effect of class imbalance on model performance, we calculated stratified metrics for each skeletal class in the test set. Table 6 presents the mean absolute error (MAE), mean squared error (MSE), and classification accuracy for Classes I, II, and III. Skel-Net achieved high predictive performance in Class I and Class III, with MAE values of 0.97° and 0.90° and accuracy rates of 79.2% and 89.2%, respectively. In contrast, Class II showed a higher MAE of 2.01° and a lower accuracy of 33.3%, which may be attributed to its limited sample size (*n* = 9). These findings provide a more granular view of class-specific model performance beyond confusion matrices and help contextualize Skel-Net’s strength in well-represented classes, while underscoring challenges in underrepresented ones.

**Table 6:** Class-wise prediction performance of Skel-Net on the test set

Class	Support (*n*)	MAE (degrees)	MSE (degrees)	Accuracy (%)
Class I	24	0.970	1.728	79.2
Class II	9	2.005	5.565	33.3
Class III	65	0.903	1.292	89.2

**Fig. 6:**
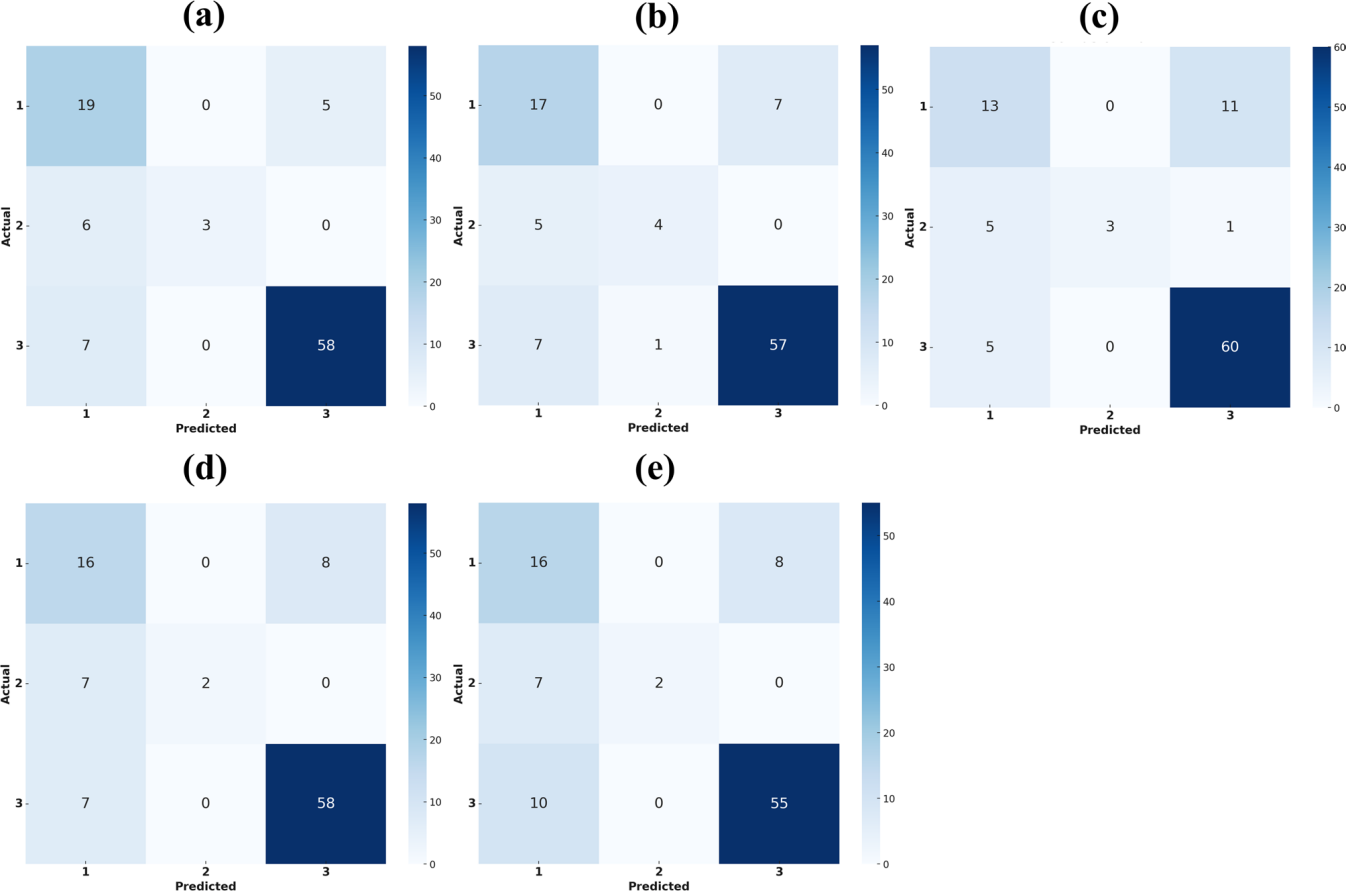
Confusion matrix for skeletal malocclusion classification using different models on lateral cephalograms. **a** Skel-Net. **b** DenseNet121. **c** MobileNetV2. **d** ResNet101. **e** VGG16

Figure 6 presents confusion matrices for the prediction performance of the various models concerning skeletal malocclusion classification. The matrices present a detailed overview of each model’s ability to correctly assign the three skeletal classes (class I, class II, and class III), highlighting true positive, false positive, and false negative values for each category. Skel-Net had a notably strong performance across all three classes, especially in correctly predicting class III, the most represented class in the test data.

Of the five models, Skel-Net had the best overall performance, excelling in the accurate prediction of class III, with 58 of 65 samples correctly identified. Skel-Net had lower misclassification rates for classes I and II, showcasing its robust ability to distinguish between skeletal features effectively. In comparison, DenseNet121 performed well but fell slightly short of Skel-Net’s accuracy, particularly in distinguishing between classes I and II. However, it achieved a strong classification rate for class III with 57 correct identifications. Although computationally efficient, MobileNetV2 performed only moderately, particularly in class I, misclassifying many samples as class III. ResNet101 performed comparably to DenseNet121, with a strong classification rate for class III but noticeable confusion between classes I and II.

**Fig. 7:**
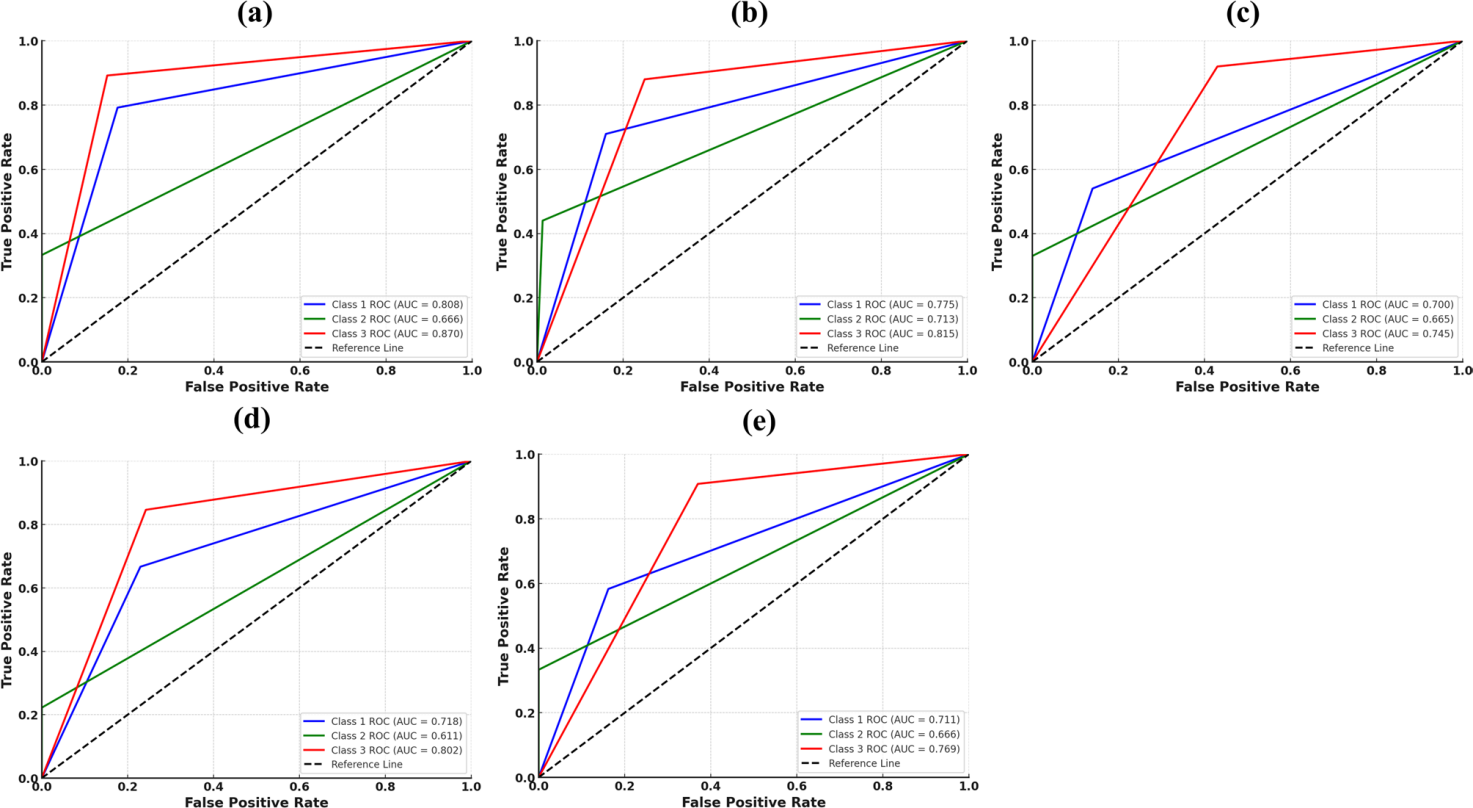
Receiver operating characteristic (ROC) curves for skeletal malocclusion classification based on lateral cephalograms. **a** Skel-Net. **b** DenseNet121. **c** MobileNetV2. **d** ResNet101. **e** VGG16. AUC, the area under the ROC curve

Figure 7 presents the receiver operating characteristic curves evaluating the ability of each model to discriminate skeletal malocclusion patterns. Skel-Net achieved the highest area under the curve (AUC) values across all classes, including an AUC of 0.87 for class III, reflecting excellent sensitivity and specificity. The receiver operating characteristic curves for Skel-Net demonstrated clear separation from the reference line across all classes, underscoring its ability to minimize false positive rates while maintaining high, accurate, favorable rates. AUC values for other models were lower, especially for classes I and II, indicating weaker performance in distinguishing those classes.

## Discussion

Understanding craniofacial growth and development is critical for accurate diagnosis and optimal orthodontic treatment planning. Predicting skeletal growth trends in children and adolescents is particularly challenging because of the complex interplay of genetic, environmental, and functional factors. Recent advances in machine learning and deep learning have shown promise for addressing those challenges. However, existing studies have often failed to develop comprehensive insights into craniofacial growth’s dynamic and longitudinal nature. To address that gap, we propose Skel-Net, an anatomic prior-guided deep learning network that predicts five-year changes in the ANB angle for children 8–16.

Longitudinal research on individual data provides critical insights into craniofacial development that static cross-sectional studies cannot achieve and is indispensable for understanding growth trajectories, particularly in orthodontics. However, such studies remain rare given the significant challenges of long-term follow-ups, resource-intensive data collection, and ethics considerations, particularly in pediatric populations [31].

The use of longitudinal data from individual patients was a central feature of the present study, offering a comprehensive perspective on craniofacial growth. Such data capture dynamic changes in craniofacial structures over time, particularly the varying growth patterns of the maxilla and mandible during key developmental periods [10]. As Fig. 1 illustrates, a temporal perspective allows individual growth trajectories to be tracked over five-year intervals, providing essential information for tailoring orthodontic treatment plans to specific patient needs. Unlike static measurements, which offer only a snapshot of a patient’s condition, longitudinal data reveal trends and variations critical for improving the reliability and accuracy of predictions [10, 32].

Notably, the collection and use of longitudinal data in pediatric populations pose significant challenges. Maintaining consistent follow-ups over extended periods requires considerable institutional resources and commitment. Additionally, ethics considerations related to repeated imaging in growing children present further obstacles, particularly given the need to minimize radiation exposure from lateral cephalograms [31]. Those challenges have contributed to the limited availability of longitudinal datasets in the field. Nevertheless, in this study, we successfully used longitudinal data to train and validate Skel-Net, demonstrating the feasibility and value of such an approach in advancing craniofacial growth research.

Our deep learning model was trained using longitudinal data from patients who underwent no interventions during the growth process. The insights into the natural development of craniofacial growth in children and adolescents thus gained will assist in devising more personalized and timely treatment plans, considering each individual’s unique growth pattern [31].

The ANB angle is a crucial cephalometric measurement extensively used in orthodontics to assess the sagittal relationship between the maxilla and mandible. It offers a straightforward and reliable diagnostic tool for identifying skeletal malocclusions, whose high specificity, sensitivity, and ease of use make it a preferred choice among orthodontists. The validity of this parameter has been examined in numerous studies [33–36]. Comparative analyses reveal that the ANB angle outperforms measurements such as the Tau angle regarding reliability and lower error rates, reinforcing its robustness and consistency in clinical practice [37, 38]. The ANB angle is also particularly advantageous because of its simplicity of application. Unlike other cephalometric measurements that might require complex calculations or advanced imaging techniques, the ANB angle is accessible and efficient to measure, making it ideal for routine clinical use and large-scale studies. Additionally, the ANB angle has demonstrated notable prediction accuracy for post-pubertal jaw relationships, suggesting its usefulness in clinical assessments and making it a valuable parameter for evaluating future jaw relationships [35].

Kim et al. [34] reported that the ANB angle is a reliable parameter for assessing anteroposterior skeletal relationships, even in growing patients with varying skeletal patterns. Their study highlighted that, despite being influenced by jaw rotation and vertical growth patterns, the ANB angle remains a robust diagnostic tool given its predictive accuracy and clinical relevance.

Beyond the quantitative results, this study provides important methodological and clinical contributions to the field of orthodontic AI. By training on longitudinal data from children and adolescents, the model reflects real-world growth patterns rather than single-time snapshots. Previous studies have primarily relied on cross-sectional datasets or single-time-point cephalograms, which fail to capture the dynamic, individualized nature of craniofacial growth [9]. In contrast, our approach incorporates longitudinal follow-up data and anatomical priors, offering a more temporally and structurally informed prediction framework. The two-stage architecture, combining landmark heatmaps and ANB priors as multichannel inputs, enhances anatomical sensitivity and supports more biologically informed learning. Moreover, the ability to predict the ANB angle as a continuous value over five years, instead of assigning categorical labels, offers clinicians time-based insights that can guide early interventions and improve long-term outcomes. Collectively, these characteristics reinforce the potential of Skel-Net as a clinically applicable and biologically grounded model for long-term skeletal growth prediction.

Recent studies have increasingly explored AI-based predictions using serial cephalograms, though the targets and intervals of prediction vary. Larkin et al. [11] applied a CNN model to predict cephalometric landmark positions over a two-year interval in skeletal Class I preadolescents, achieving acceptable accuracy for most hard-tissue points but limited performance for chin-related soft-tissue landmarks. Compared to their approach, our model extends the prediction window to five years and targets the clinically interpretable ANB angle, enabling direct inference of skeletal class. Furthermore, the use of multichannel inputs combining heatmaps with anatomical priors enhances prediction robustness. Additionally, a recent review by Neeraja et al. [39] emphasized the importance of anatomically informed and interpretable architectures in AI-driven orthodontic tools. Our prior-guided design aligns with this recommendation, embedding anatomical meaning directly into the learning framework.

In parallel, Cicek et al. [40] proposed a novel methodology for orthodontic growth stage detection based on chaotic functional connectivity matrices and fractal dimension analysis, demonstrating the diversity of AI applications in developmental assessment. While their approach focused on neurofunctional signals, Skel-Net contributes a structurally grounded alternative that leverages routinely acquired lateral cephalograms, offering a clinically accessible and biologically interpretable model for long-term growth prediction.

As summarized in Table 2, Skel-Net outperformed other models across both regression (MAE: 1.021°, RMSE: 1.338°, R²: 0.517) and classification metrics (accuracy: 81.6%, precision: 83.8%). This dual strength is further illustrated in Fig. 6, where the confusion matrix highlights Skel-Net’s reliable discrimination across skeletal classes, particularly its high accuracy in classifying class III cases. This consistent outperformance across multiple deep learning backbones—including DenseNet121, MobileNetV2, ResNet101, and VGG16—suggests that Skel-Net is robust to architectural variation (Table 2). Its superiority in both regression and classification tasks, despite differences in model design, implies strong generalizability across diverse input conditions.

While explicit noise-based robustness tests were not performed, the model’s stable performance across structurally distinct networks and cephalometric inputs indirectly supports its resilience in real-world clinical settings. The integration of heatmap-guided anatomical priors may contribute to this robustness by reinforcing biologically meaningful features during training.

Moreover, Fig. 7 shows that Skel-Net achieved the highest area under the ROC curve (AUC), supporting its sensitivity and specificity in clinical classification. These aligned outcomes suggest that Skel-Net’s fine-grained ANB angle predictions contribute meaningfully to categorical classification performance, reinforcing its potential utility as a clinical support tool for skeletal growth assessment.

From a clinical perspective, even minor improvements in predictive accuracy can inform the timing and approach of orthodontic interventions, particularly in borderline cases where early treatment decisions are critical. In particular, Skel-Net may be useful for early identification of borderline skeletal patterns, where growth predictions could guide clinicians toward timely and potentially less invasive treatments. Thus, Skel-Net provides valuable support for personalized treatment planning. The Bland–Altman plots in Fig. 5 further corroborate this predictive consistency, which shows narrow limits of agreement and minimal bias compared to competing models.

While Skel-Net demonstrated the lowest MAE among all compared models (~ 1.0°), this value should be interpreted in light of the inherent variability in ANB measurements. Del Santo Jr et al. [36] showed that variations in occlusal plane inclination can influence the ANB angle by more than 1°, and Ishikawa et al. [35] found inter-observer differences of similar magnitude. These findings suggest that Skel-Net’s prediction error lies within a clinically acceptable range. As such, the model should be regarded as a complementary aid for skeletal growth forecasting, rather than a definitive diagnostic tool.

Skel-Net’s clinical applicability is further highlighted in its ability to accurately classify skeletal malocclusion classes (Fig. 7), achieving an AUC of 0.87 for class III and outperforming the other models. The multichannel inputs to Skel-Net, including heatmaps and ANB priors, enhance its sensitivity and specificity, particularly for complex cases. In contrast, models such as DenseNet121 and MobileNetV2 had lower AUC values, indicating weaker performance.

Despite the application of a class-weighted loss function to address dataset imbalance, the performance of Skel-Net varied across skeletal classes. As shown in the stratified analysis (Table 6), prediction accuracy and error rates were stable for Class I and III, indicating the model’s effectiveness in handling the dominant and mid-sized categories. However, performance in Class II was relatively lower, with increased prediction error and reduced classification accuracy. This discrepancy likely stems from the small number of Class II samples, which may have limited the model’s ability to generalize. These results highlight the impact of data imbalance on class-specific performance, suggesting that future studies should consider alternative approaches to balance the dataset, such as synthetic augmentation or targeted data collection. Although oversampling methods like SMOTE are less directly applicable to regression targets such as ANB angles, other techniques—such as GAN-based data generation or class-conditional sampling—may offer potential improvements.

Skel-Net’s predictive capabilities enable early identification of growth patterns, facilitating timely interventions for optimized treatment outcomes. Prediction of the ANB angle over five years aids clinicians in anticipating skeletal discrepancies and planning treatments tailored to individual growth trajectories. This personalized approach can significantly reduce the need for more invasive or complex interventions later in life. Additionally, the model’s ability to provide dynamic insights into craniofacial development ensures that treatment timing aligns with critical growth periods, optimizing patient outcomes and improving long-term stability.

While the ANB angle is generally stable during the preadolescent to adolescent growth period, even minor variations can have significant implications for orthodontic diagnosis and treatment timing. Accurately predicting these subtle changes is crucial, especially in cases where early intervention can prevent the progression of skeletal discrepancies. The ANB angle remains a fundamental and accessible parameter in clinical practice for assessing sagittal skeletal relationships. This study aimed to improve the predictive accuracy of this key metric, thus supporting early diagnosis and personalized treatment planning.

While Skel-Net demonstrated strong performance, certain limitations warrant further investigation in future research. First, reliance on data from a single institution introduces the potential for selection bias, limiting the generalizability of our findings. Second, because the model was validated only on internal datasets, its applicability to external datasets featuring diverse patient populations and digital imaging formats remains untested. Expanding the dataset to include a wider age range and data from multiple institutions will improve the model’s robustness and applicability. Additionally, the model does not account for ethnic or sex-based morphological differences, which may influence craniofacial growth trajectories. The relatively narrow age range and exclusion of orthodontically treated cases may also limit the generalizability of our findings to broader clinical populations. Integrating multimodal data sources such as three-dimensional imaging or demographic information could enhance predictive accuracy and clinical relevance, and validating the model using publicly available datasets could ensure consistent performance across clinical settings, further strengthening Skel-Net’s utility.

## Conclusions

In this study, we proposed Skel-Net, a deep learning model designed to predict five-year changes in ANB angles using longitudinal cephalometric data from children and adolescents. Skel-Net outperformed baseline models across regression and classification metrics, demonstrating clinically meaningful accuracy despite moderate explanatory power (*R*² = 0.517). Its two-stage structure, incorporating anatomical priors, enabled more dynamic and personalized growth prediction. These results highlight the feasibility of using AI to support early skeletal assessment and orthodontic planning. However, limitations remain, including dataset imbalance and lack of external validation. Future work should focus on applying the model to more diverse datasets and incorporating multimodal inputs to enhance generalizability and robustness.

## Data Availability

To protect patient privacy, the datasets generated and/or analyzed during the current study are not publicly available because of restrictions placed by the Institutional Review Board of Seoul National University’s School of Dentistry. However, the datasets are available from the corresponding author on reasonable request. Please contact the corresponding author for any commercial implementation of this research.
